# Cross-Cultural, Aboriginal Language, Discovery Education for Health Literacy and Informed Consent in a Remote Aboriginal Community in the Northern Territory, Australia

**DOI:** 10.3390/tropicalmed3010015

**Published:** 2018-01-29

**Authors:** Jennifer M. Shield, Thérèse M. Kearns, Joanne Garŋgulkpuy, Lisa Walpulay, Roslyn Gundjirryirr, Leanne Bundhala, Veronica Djarpanbuluwuy, Ross M. Andrews, Jenni Judd

**Affiliations:** 1 ARDS Aboriginal Corporation, Winnellie, NT 0821, Australia; 2 Department of Pharmacy and Applied Science, La Trobe University, Bendigo, VIC 3552, Australia; 3 Menzies School of Health Research, Charles Darwin University, Darwin, NT 0811, Australia; therese.kearns@menzies.edu.au (T.M.K.); walpulay@gmail.com (L.W.); roslyn.dhurrkay@menzies.edu.au (R.G.); djarpanbuluwuy@gmail.com (V.D.); ross.andrews@menzies.edu.au (R.M.A.); 4 Yalu’ Marŋgithinyaraw, Galiwin’ku, NT 0822, Australia; yaluoffice@gmail.com; 5 School of Health, Medical and Applied Sciences, Central Queensland University, Bundaberg, QLD 4670, Australia; j.judd@cqu.edu.au; 6 College of Medicine and Dentistry, Anton Breinl Research Centre for Health Systems Strengthening, James Cook University, Townsville, QLD 4811, Australia

**Keywords:** cross-cultural health education, health education, Aboriginal language, worldview, health literacy, discovery education, informed consent, scabies, strongyloidiasis

## Abstract

Background: Education for health literacy of Australian Aboriginal people living remotely is challenging as their languages and worldviews are quite different from English language and Western worldviews. Becoming health literate depends on receiving comprehensible information in a culturally acceptable manner. Methods: The study objective was to facilitate oral health literacy through community education about scabies and strongyloidiasis, including their transmission and control, preceding an ivermectin mass drug administration (MDA) for these diseases. A discovery education approach where health concepts are connected to cultural knowledge in the local language was used. Aboriginal and non-Aboriginal educators worked collaboratively to produce an in-depth flip-chart of the relevant stories in the local language and to share them with clan elders and 27% of the population. Results: The community health education was well received. Feedback indicated that the stories were being discussed in the community and that the mode of transmission of strongyloidiasis was understood. Two-thirds of the population participated in the MDA. This study documents the principles and practice of a method of making important Western health knowledge comprehensible to Aboriginal people. This method would be applicable wherever language and culture of the people differ from language and culture of health professionals.

## 1. Introduction

Australian Aboriginal peoples, especially those who live in remote communities, experience a higher burden of disease, more than three times that of the Australian national average [1]. Most lack the knowledge about how to be healthy when living a Western lifestyle [2].

One of the factors contributing to the high burden of disease is limited health literacy [3,4]. People with low health literacy have a higher risk of poorer health outcomes [4,5]. The Australian Commission on Safety and Quality in Health Care [6] defines individual health literacy as ‘the skills, knowledge, motivation and capacity of a person to access, understand, appraise and apply information to make effective decisions about health and health care and take appropriate action’, and this definition is modified from Sørenson’s [7] model of health literacy. This definition fits Nutbeam’s concept of ‘critical health literacy’ [8] and assumes good literacy skills. However, Nutbeam, referring to the work of Freire [9], indicated that outcomes similar to critical health literacy could be achieved by those with low or no skills in reading and writing through interpersonal forms of communication and community-based health education [8]. Similarly, Baker [10] divides health literacy into health-related print literacy and health related oral literacy, the ability to orally communicate about health. In the context of Aboriginal people living remotely, health related oral literacy is achievable. 

Both language and culture (including worldview) act as barriers for Aboriginal people in achieving health literacy in the Western cultural context, especially for those living remotely [11]. Worldview, the way people construct meaning and understand the world around them [12], is closely related to language and culture. Aboriginal languages, cultures, and worldviews are very different from the English language and Australian non-Aboriginal culture and worldview [12,13]. Aboriginal worldviews supported them adequately when living a traditional lifestyle. The difficulty arises because the traditional roles of doctor and midwife have been taken over by non-Aboriginal health professionals with a different worldview [14], so people need the necessary cross-cultural understanding to do well when in need of medical assistance [14,15].

Essential general knowledge about germs, blood circulation, immunity, and the cellular composition of the body, foundational for Western understanding about health, is not part of the general knowledge of Aboriginal people living remotely [11,14]. One group of Aboriginal people recognised that the reason that they were unable to understand information in the English language was because of missing information that they called ‘the secret English’ [13]. ‘The secret English’ was in fact general knowledge for their English-speaking informants but not for them. Some Aboriginal people even believe that key information about their sickness is deliberately hidden [15]. So when informing people about their health, relevant foundational knowledge, as well as the specific information, is needed for understanding to occur.

An essential ingredient for effective communication and health literacy is the provision of comprehensible information [4,6]. For Aboriginal and Torres Strait people to achieve health literacy, ‘it is necessary for information to build on Indigenous understandings and perspectives’ [6]. Aboriginal people have cultural knowledge such as the organs of the body and the behaviour and life cycles of food animals and plants that can be used as starting points to connect new health information to what they already know [14]. 

Aboriginal people can become health literate about a particular topic by engaging in community education when the knowledge is shared in their language, is connected to their cultural knowledge, is directed to the whole community, and is shared in a culturally sensitive manner [11,12,14,16]. For new information to become part of the cultural knowledge of an individual, it is essential that the information is accepted by the cultural group as a whole, so that it can be maintained, updated, and passed on to the next generation. This can be achieved when the majority of adults in the cultural group hear, understand, and discuss the new information [14].

This style of education, first documented in 2000 [14] and later called discovery education [16], is also known as story methodology [17]. It was developed to help Yolŋu Aboriginal people of north-east Arnhem Land understand law, economics, and health. Discovery health education uses a collaboration between non-Aboriginal and Aboriginal health educators to find connections between the health information and existing cultural general knowledge and to develop a cross-cultural story in the local language that is shared with the community.

In this study, community health education was undertaken in a remote community using a discovery education approach that focussed on the diseases scabies (caused by mites, *Sarcoptes scabei*) and strongyloidiasis (caused by roundworms, *Strongyloides stercoralis)* and the effect of treatment. The education was part of the preparation for a research project examining the effect of ivermectin mass drug administration (MDA) on the two diseases [18,19], so that potential participants could give informed consent and learn how to prevent the diseases. The education aimed to facilitate the development of health literacy in these two diseases so that in the future, the participants would be able to connect further information about infectious diseases to the foundational knowledge obtained from this education process.

## 2. Materials and Methods

### 2.1. Population

The health education took place in Galiwin’ku, a remote Aboriginal community with a population of approximately 2000, located 550 km east of Darwin, Northern Territory (NT), Australia. This project received ethical approval from the Human Research Ethics Committee of the NT Department of Health and Menzies School of Health Research (EC00153—project 09/34).

### 2.2. Study Objective and Evaluation

The study objective was to facilitate the development of oral health literacy through community education about the nature of scabies and strongyloidiasis, how these diseases are transmitted, and how to prevent and treat these diseases.

Expected outcomes of the education were:

- an informative cross-cultural flipchart in Djambarrpuyŋu language about scabies and strongyloidiasis, how they are transmitted, and how to prevent and treat these diseases, developed in collaboration with non-Aboriginal and Aboriginal educators;
- non-Aboriginal and Aboriginal educators conducting the education together in a conversational style;
- discussion of the educational stories by the people amongst themselves;
- feedback from community members indicating that aspects of the story are understood;
- a large number of people participating in the ivermectin MDA project.

### 2.3. Consultation with Clan Elders

Community-based workers facilitated informal discussions with the community elders, who gave their approval for the project. 

### 2.4. Community Health Education Principles

The community health education followed the principles of discovery education (Table 1). Discovery education uses a culturally acceptable process and employs a collaborative approach between non-Aboriginal health educator/s and Aboriginal educator/s. The non-Aboriginal educators provide the information and have some knowledge of the language and culture, and the Aboriginal professionals know some English and have an in-depth knowledge of their language, culture, and worldview. Educational stories are developed by connecting health concepts to cultural knowledge in the local language. 

### 2.5. Development of Educational Materials

A flip-chart, information sheet, and consent form were prepared in Djambarrpuyŋu language, the local *lingua franca*, and in English. These flip-charts contained details about scabies mites, *Strongyloides* worms, and the research project. They incorporated existing educational resources [20,21,22,23,24,25] as well as components developed especially for the project. The traditional story about cycad food (*Cycas armstrongii*) was used as an analogy for the project. This story belongs to the Wangurri Clan of north-east Arnhem Land, and an elder of this clan ‘an owner of the knowledge’ recorded the story and gave permission to use it for the project. This story about how to collect cycad nuts, leach out the poison in running water and prepare a safe, delicious, and nutritious food, is well-known to Yolŋu people. This story was compared with eliminating scabies and *Strongyloides* from the community by washing hands, wearing shoes, stopping taps from leaking to keep the ground dry, keeping the house clean, washing clothes and airing bedding in the sun, showering, using the toilet and keeping it clean, giving faecal and/or blood samples for testing for *Strongyloides*, and taking the medicine to eliminate scabies and *Strongyloides* from the body. 

The stages in the life cycle of *Strongyloides* were likened to the stages in the human life cycle. The rhabdiform larvae that exit the body via the faeces were called *Strongyloides* djamarrkuli’ (*Strongyloides* children). *Strongyloides* infective larvae that can invade the body are all immature females, so were called ‘wirrkul*Strongyloides’* or ‘wirrkul mewirri’ (pre-pubescent female *Strongyloides* or pre-pubescent female worms). The parasitic adults were called ‘ŋäṉḏi *Strongyloides*’ (mother *Strongyloides*) because they are all female.

Similarly, the stages of the life cycle of scabies were compared with the human life cycle. The name given to scabies was ‘scabies-puy dhirrkthirrk’ or scabies itch. Illustrations included scanned drawings by the Aboriginal educators, photographs prepared by the team, and photographs and drawings from pre-existing educational materials in English. 

**Figure 1:**
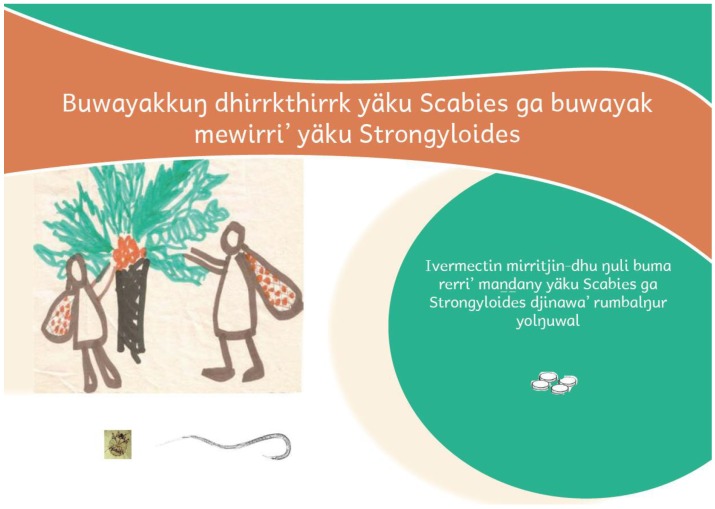
Title page of the flipchart. The title translates as ‘Eliminating scabies mites and invisible *Strongyloides* worms’. The subtitle: ‘Ivermectin medicine kills both diseases in people’s bodies’. The title page includes symbols of the main components of the story: the overall analogy (the cycad story), a scabies mite, an adult female *Strongyloides* worm, and the ivermectin tablets.

The Djambarrpuyŋu text and illustrations were assembled in a power-point presentation after each flip-chart preparation session and reviewed at the beginning of the next session. When complete, an English version was prepared, and the final formatting of both flip-charts was done by a graphic designer. The final files were printed, laminated and assembled into a flipchart (Figure 1) and are available at the following web addresses: https://www.menzies.edu.au/icms_docs/162089_Mites_and_worms_flipchart_English_version.pdf; https://www.menzies.edu.au/icms_docs/162094_Mites_and_worms_flipchart_Yolngu_version.pdf.

### 2.6. Presentation of the Story to the Elders of the Community

The first educational session took place at an indoor meeting of the *Dhuni Forum*, a group comprising representative elders of each clan. The project was explained to them in English, and details of scabies and strongyloidiasis were explained in Djambarrpuyŋu language.

### 2.7. Carrying out Scabies and Strongyloidiasis Education

Scabies and *Strongyloides* health education took place over eight weeks in February–March 2010, prior to the commencement of the MDA project. Two community education teams were formed, one male and one female, so that men could talk with men and women with women, an important cultural issue for more traditional Aboriginal communities. Each team included a non-Aboriginal health educator and one or more Aboriginal educators. The teams visited households and work places as people were available, and engaged with either single sex groups, or family groups. Before each education session took place, usually the previous day, Aboriginal staff visited households to find a convenient time for the education. Most often, the education took place out-of-doors, on a veranda or under a tree. 

### 2.8. Supplementary Materials

The flip-chart was supplemented by pictures of microscopes and microscope movie clips of a scabies mite, rhabitiform *Strongyloides* larvae, filariform *Strongyloides* larvae, and bacteria (responsible for secondary infection) [23]. The educational DVDs on scabies [21] and *Strongyloides* [22] were also used occasionally. An information sheet about the project in Djambarrpuyngu language (or occasionally in English if more appropriate) was left with each household.

## 3. Results

### 3.1. Flipchart

The front page of the flipchart is shown in Figure 1, and the topic of each page of the flipchart is listed in Table 2. 

### 3.2. Community Education

The stories from the flip-chart were shared with people from 111 houses, 70% of the houses in the community (*n* = 159 at the time of the education [18]). At 41 houses (37% of those visited) either the men’s team or the women’s team spoke with family groups. At another 43 houses (39%) the men’s team spoke to men only, and at 27 houses (24%) the women’s team spoke to women only. This is the culturally acceptable way of educating the community. In addition, two homelands (small outlying settlements), eight workplaces, and two schools were visited. The number in each age group as percent of the estimated 2011 population and sex are given in Table 3. The people who took part in the education were predominantly in the 30–49 years age group.

Most of the people were very interested in the stories and asked questions. The movie clips were particularly effective in giving people an understanding of disease-causing organisms, foundational to understanding scabies and *S. stercoralis* infection. The role of faecal material from infected people in the transmission of *S. stercoralis* also became clearer to people when they saw the movie clips. Children enjoyed watching the *Strongyloides* Story DVD.

### 3.3. Community Discussion of the Stories

People were fascinated by the idea of the ‘wirrkul mewirri’ (pre-pubescent female worm), the infective filariform larva. The story of the wirrkul mewirri spread through the community. On a number of occasions, teenagers passing by called out ‘wirrkul mewirri’ to the non-Aboriginal educator, as they passed her in the street. The story also spread to the city through children who had visited the community and then told their father when they went home. Their father then wanted to know more. *Strongyloides* became known locally as ‘wirrkul mewirri’. People who had missed out on hearing the stories about scabies and *Strongyloides* were asking the Aboriginal educators why the education had stopped. In 2017, a person who was being shown gut bacteria in a movie clip that also included *Strongyloides* worms asked whether the worms were ‘wirrkuḻ mewirri’, demonstrating that the introduced term has currency seven years after it was introduced.

### 3.4. Feedback Indicating that the Mode of Transmission of Strongyloides Was Understood

Children were overheard telling each other to be careful where they put their feet, for fear of the wirrkul mewirri. Teenagers were overheard teasing each other when not wearing footwear, and telling each other that the wirrkul mewirri would get into them. One of the non-Aboriginal teachers at the school offered to do some shopping for her Aboriginal neighbours while she was away on leave. They asked her to bring thongs, footwear for all the family, to protect them from the wirrkuḻ mewirri, indicating that the mode of transmission was understood.

### 3.5. Participation in the Ivermectin MDA

A large proportion of the population participated in the MDA research project. In 2010, there were 1013 participants, and in 2011 an additional 360 people joined the project, a total of 1373 [18,19], approximately 65% of the 2011 estimated population [26].

## 4. Discussion

This study fills a gap in the literature by documenting the principles and practice of discovery education, an effective method of cross-cultural community education that uses a collaborative approach to make important information in the Australian western context comprehensible to Aboriginal Australians living remotely. In the Australian context, there is no generally accepted effective strategy to promote health literacy in Aboriginal people who retain their worldview and language. There are few studies reporting effective community health education in the literature [11,12,16,17]. This paper outlines an effective process of co-constructing narrative health education materials, and of face-to-face communication of community health information to improve knowledge, health literacy, and health outcomes of the community.

The large number of people who participated in the ivermectin MDA project in 2010, and the substantial number of additional people who participated in 2011, suggests that the community education described above was effective in informing people about scabies and strongyloidiasis and the benefits of the MDA. The process also helped them take a step towards achieving oral health literacy about the causes of scabies, strongyloidiasis, and infectious diseases and the benefits of treatment for these diseases.

The educational process followed the principles of discovery education (Table 1). The education was acceptable to people because the education teams included a non-Aboriginal educator, an ‘owner of the knowledge’, and even when the story was being told by an Aboriginal educator, it was recognised to be on behalf of the non-Aboriginal educator, who was available to answer questions and clarify any points not understood. The education was given in a ‘culturally correct way’, having been first approved by the respected clan leaders, and it was provided in Djambarrpuyŋu language, the local *lingua franca*, which is readily understood by everyone in the community. It covered foundational information, including microscopic organisms and cells of the body, and it was connected to cultural general knowledge, essential for understanding the life cycles of scabies mites and *Strongyloides* worms and how they affect the body. It connected important behaviours for preventing the transmission of scabies and strongyloidiasis with a cultural story, the preparation of cycad food. Thus, the information was built on Indigenous understandings and perspectives [6] (p. 22).

Interpersonal community health education in remote communities can be very effective because of the strong kinship network in these communities [27]. The kinship network is also the basis for social interactions. When information is given to a large number of people in the community, it becomes a topic of conversation. This gives people an opportunity to debate the information and decide whether it makes sense and is important to them. Feedback to the education team indicated that intellectual debate about the health stories was occurring in the community. It is likely that the information was able to survive intellectual debate. 

The partnership between non-Aboriginal educators and Aboriginal educators was crucial to the success of the education. It enabled consultation with the clan elders, the preparation of comprehensible stories, and the sharing of the stories in a conversational style that encouraged the learners to ask questions and find out what they wanted to know. The stories were accurate and told in a way that provided a basis for understanding important health principles, especially the causes of infectious diseases. This foundational understanding has the potential to help people make sense of new health information in the future.

To what extent the knowledge has been incorporated into the cultural knowledge of the group, that is, whether it has been accepted, maintained, updated and re-taught by the group, is not known at this stage. Approximately 31% of people 15 years and over were directly reached by the education teams, whereas Trudgen [14] (p. 210) suggests that peer group affirmation of the majority of the adult population is needed in order for the information to become part of the cultural knowledge of the group. However, the 2017 query about the wirrkul mewirri is a positive sign that at least some of the knowledge is retained.

Although the discovery education method involves Aboriginal and non-Aboriginal people learning from each other, it is *not* the ‘two-way education’ that Harris [13] (p. 129) proposed for school education. He recommended that children be educated simultaneously but separately in cultural knowledge of their own people in their Aboriginal language, and Western knowledge in English. In discovery education, connections between the Western knowledge and Aboriginal general knowledge are explored so that people can incorporate the Western knowledge into their existing cultural general knowledge.

In recent years, there has been increased awareness of the importance of principles of adult learning and education for critical consciousness in primary health care for remote-living Aboriginal people [28], but the use of the local language as an integral part of the education process is not mentioned, and the use of the discovery education principles is rare. Although Australia’s *Translating and Interpreting Service* offers the most extensive telephone interpreting system in the world, this service does not include Indigenous languages [29].

The importance of Aboriginal languages in communicating health information cannot be overemphasised. It is necessary but not sufficient for non-Aboriginal health professionals to be trained in Aboriginal culture and in cross-cultural communication skills [15]. A knowledge of the language is also needed to facilitate health literacy in Aboriginal people living remotely. Although all NT public sector agencies are required to provide cross-cultural awareness training to all employees who interact with Aboriginal people, including health professionals [30], they do not recognise the importance of language. The NT Health Promotion Framework [31] includes language barriers as one of the factors that compound the negative effect of low socioeconomic status on health, but it does not address the need to train specialised non-Aboriginal health professionals to become proficient in an Aboriginal language. 

## 5. Conclusions

This study provides an example of the ability of a partnership of non-Aboriginal and Aboriginal health educators using a discovery education approach to develop and share a cross-cultural health story that people understood, in an Aboriginal language. The study also provides a model for community health education to facilitate the development of health literacy in Aboriginal people living remotely.

A discovery education approach to health education would be useful in any part of the world where the language and worldview of the majority of the people are different from that of health professionals.

## Figures and Tables

**Table 1:** Principles of Discovery Education (Modified from Trudgen [14] (pp. 202–210).

Principle	Explanation
The educator/s are credible in the eyes of the people	Traditional Yolŋu knowledge is owned by particular clans and clan elders and only they have the authority to share it. Knowledge of modern diseases is considered by Yolŋu to be ‘owned’ by non-Aboriginal health professionals because Yolŋu people consider that these diseases are of European origin.
The educator/s follow ‘culturally correct’ steps for providing the information	The new information is presented first to the clan elders for their approval, and then shared with the whole cultural group, particularly the adults.
The information is provided in the local language	The majority of remote-living people have only a superficial knowledge of English. They can draw on sophisticated concepts in their own language to help them understand relevant health information.
The information is built on culturally accepted knowledge and truths	This involves searching for key terms and stories in the local language, and using ways, such as analogy, of connecting the new knowledge with cultural general knowledge.
The educators use a dialogue style [ 9]	The educator and learner are learning from each other. This enables the educator to clarify information that is not clear and to provide answers to what the learners want to know.
The information is rigorous and in-depth	Comprehensible information that can survive intellectual debate is accepted. If it is ‘simplified’ or superficial, it is rejected.

**Table 2:** Contents of the educational flipchart for the ivermectin mass drug administration (MDA) project.

Page	Contents
Title	Summary of the aim of the ivermectin research project and the effect of ivermectin ( Figure 1)
1	Brief summary of the traditional story of preparing food from cycad nuts as an overall analogy for the project
2	Diagram illustrating what people can do to eliminate scabies and *Strongyloides*
3	The role of the microscope in making it possible to see bacteria, *Strongyloides* worms, and scabies mites
4	Good and bad bacteria and secondary infection
5	Immunity, focusing on the role of white cells
6	Immunity, focusing on the role of antibodies
7	Direct life cycle of *Strongyloides*, emphasizing the role of parasitic adults in reproduction, of larvae in transmission via the faeces, and immature female infective larvae in entering the body through the skin
8.	*Strongyloides* autoinfective cycle, implications for life-long infection, and overwhelming infection when the white cells cannot do their work; an assurance that ivermectin can kill *Strongyloides* in our body
9	Secondary infection occurs when *Strongyloides* larvae enter the body proper through the wall of the lower gut, accompanied by bacteria
10	Symptoms of strongyloidiasis
11	Life cycle of scabies mites
12	Symptoms of scabies
13	Secondary infection associated with scabies
14	Transmission of scabies, mainly by person-to-person contact
15	Ivermectin treatment program: testing for scabies and strongyloidiasis, and medication for different age groups
16	Ivermectin treatment program: taking medicine, following-up at 6 months, repeating after 1 year, following up again at 18 months, and informed consent
17	A detailed version of the cycad story
18	Acknowledgements
Back page	Summary of what people can do to eliminate scabies mites and *Strongyloides* worms from their bodies

**Table 3:** Age groups as percent of estimated population and sex of people who took part.

Age Group	Estimated Population [26]	Total Seen (% of pop.)	% Male
3–14 years	754	148 (20)	45
15–29 years	541	115 (21)	63
30–49 years	568	254 (45)	51
50+ years	260	62 (24)	47
Total	2123	579 (27)	51
